# Cardiopulmonary involvement in juvenile systemic sclerosis: development of recommendations for screening and investigation

**DOI:** 10.1186/1546-0096-12-S1-P150

**Published:** 2014-09-17

**Authors:** Clare Pain, Tamas Constantin, Eileen Baildam, Christian Beyer, Henning Lenhartz, Michael Blakley, Dana Nemcova, Clarissa Pilkington, Ivan Foeldvari

**Affiliations:** 1 Pediatric Rheumatology, Liverpool, UK; 2 Pediatric Rheumatology, Budapest, Hungary; 3 Pediatric Rheumatologs, Liverpool, UK; 4 Pediatric Cardiology, Hamburg, Germany; 5 Pediatric Gastroenterology, Hamburg, Germany; 6 Pediatric Rheumatology, Indiana, USA; 7 Pediatirc Rheumatology, Prague, Czech Republic; 8 Pediatric Rheumatology, London, UK; 9 Hamburger Zentrum für Kinderrheumatologie, Hamburg, Germany

## Introduction

There are currently no agreed recommendations on how to investigate children for cardiopulmonary involvement in Juvenile Systemic Sclerosis (JSSc). The aim of screening is to detect disease early to facilitate early aggressive therapy and improve outcomes. Cardiopulmonary involvement is the leading cause of death in JSSc and cardiopulmonary at diagnosis incurs a worse outcome [1]. Most deaths occur early in the disease course [1, 2].

## Objectives

To develop recommendations for investigation of cardiopulmonary in JSSc, based on paediatric evidence and where this was lacking, consensus expert agreement.

## Methods

Members of the PRES Scleroderma Working Group were invited to participate; additionally a paediatric cardiologist was invited. A nominal group technique was used. 75% consensus was defined as agreement.

## Results

Table 1 shows the recommendations for screening for cardiopulmonary at baseline and at defined time points from diagnosis. Other recommendations agreed by the group which are relevant at any stage in the disease course are as follows (in Table 1).

**Table 1:** Recommendations for screening for cardiopulmonary involvement in JSSc at baseline and follow-up (75% consensus defined as agreement)

**Cardiopulmonary**	**Baseline**	
	
	All patients should undergo: - BP - 12 lead ECG - 24 hour ECG - ECHO with Doppler - Cardiac MRI with gadolinium - HRCT thorax - PFT with DLCO - 6MWT	
	
	Follow-up screening (for first 5 years from diagnosis)*	
	
	6 monthly	12 lead ECG ECHO with doppler 6MWT PFT with DLCO
	
	Annual	24hr ECG
	
	At 3 years	Repeat HRCT

*screening guidelines are based on asymptomatic patients. However, children may need more frequent monitoring depending on clinical status and abnormalities detected on previous investigation.

Recommendations are based on low grade evidence and in the most part from expert consensus opinion with extrapolation from adult studies.

## Conclusion

JSSc has a significant mortality particularly early on in the disease course. The objective of an aggressive screening program is to identify cardiopulmonary involvement at a stage which may be amenable to treatment. The recommendations developed by this group aim to standardise care and improve outcomes in this rare disease.

## Disclosure of interest

None declared

## Abbreviations

BP: blood pressure; ECG: electrocardiogram; ECHO: echocardiogram; MRI: magnetic resonance imaging; HRCT: high resolution computerised tomography; PFT DLCO: pulmonary function tests with diffusion capacity of lung for carbon monoxide; 6MWT: 6 minute walk test; NT BNP: *N-terminal pro*-*brain natriuretic peptide; GORD: gastro-oesophageal reflux disease*.

